# Impact of trauma exposure and depression comorbidity on response to transdiagnostic behavioral therapy for pediatric anxiety and depression

**DOI:** 10.1038/s44184-023-00049-4

**Published:** 2024-02-27

**Authors:** Felix Angulo, Pauline Goger, David A. Brent, Michelle Rozenman, Araceli Gonzalez, Karen T. G. Schwartz, Giovanna Porta, Frances L. Lynch, John F. Dickerson, V. Robin Weersing

**Affiliations:** 1 Department of Psychology, San Diego State University, San Diego, CA USA; 2 SDSU/UC San Diego Joint Doctoral Program in Clinical Psychology, San Diego, CA USA; 3 University of Pittsburgh School of Medicine, Pittsburgh, PA USA; 4 UPMC Western Psychiatric Hospital, Pittsburgh, PA USA; 5 Department of Psychology, University of Denver, Denver, CO USA; 6 Department of Psychology, California State University Long Beach, Long Beach, CA USA; 7 Children’s Hospital of Philadelphia, Philadelphia, PA USA; 8 Kaiser Permanente Center for Health Research, Portland, OR USA

**Keywords:** Psychology, Paediatric research

## Abstract

By adolescence, two-thirds of youth report exposure to at least one traumatic event, yet the impact of trauma history is not routinely considered when evaluating the effect of psychotherapeutic interventions. Trauma may be a particularly important moderator of the effects of transdiagnostic therapies for emotional disorders, as trauma exposure is associated with risk for the development of comorbid depression and anxiety. The current study examined the history of trauma exposure and the presence of clinically significant depression as moderators of treatment outcomes in the Brief Behavioral Therapy (BBT) trial, the largest study of transdiagnostic psychotherapy for youth. Youths (age 8–16 years) were randomized to BBT (*n* = 89) based in pediatric primary care or assisted referral to outpatient community care (ARC; *n* = 86). Clinical response, functioning, anxiety symptoms, and depression symptoms were assessed at post-treatment (Week 16) and at follow-up (Week 32). A significant three-way interaction emerged between the treatment group, comorbid depression, and trauma exposure. BBT was broadly effective for 3/4 of the sample, but, for anxious-depressed youth with trauma exposure, BBT never significantly separated from ARC. Differences in outcome were not accounted for by other participant characteristics or by therapist-rated measures of alliance, youth engagement, or homework completion. Implications for models of learning and for intervention theory and development are discussed.

## Introduction

While anxiety and depression are the most common mental health conditions in youth, they are among the least likely to receive treatment^1^ and interventions that are routinely delivered in practice are unlikely to meet best-practice guidelines^2^. Concurrent and longitudinal comorbidity between anxiety and depression is high, likely due to shared mechanisms of etiology and maintenance^3^. In recent years, transdiagnostic psychotherapies targeting these shared mechanisms have been developed, with the hope that the efficiency of such interventions may increase the dissemination of evidence-based therapy for these under-treated disorders. Note that these transdiagnostic psychotherapies differ from modular or algorithmic treatment approaches^4^ in that the same core treatment elements are delivered to all youths, simplifying implementation. The transdiagnostic psychotherapy literature in youth is still small; however, initial randomized controlled trials (RCT) have resulted in improvements in diagnostic severity, rate of remission, and functional impairment at post-treatment, across several independent investigative teams^5–9^.

While these results appear quite promising, the question of whether these psychotherapies are truly transdiagnostic remains open. Studies have reported variable effects on anxiety versus depression *outcomes*^7,10,11^, and the level of comorbidity between anxiety and depression at the start of treatment remains an underexplored *moderator* of transdiagnostic treatment effects, investigated in only one trial^9^. Further, the existing literature has yet to explore the potential impact of youth exposure to trauma on the outcomes and processes of transdiagnostic therapy, despite substantial evidence that rates of trauma exposure in youth are extremely high^12^ and are specifically associated with risk for developing comorbid depression and anxiety in youth^13,14^.

While the diagnosis of post-traumatic stress disorder (PTSD) in youth is not widely prevalent (4.7%)^15^, exposure to traumatic events is. Over two-thirds of adolescents report exposure to at least one traumatic event by age 16, such as physical or sexual abuse, being a victim to or witnessing a violent crime, or being involved in a car accident that caused injury^12^. Childhood trauma exposure incurs an increased risk of developing depression and anxiety^16–18^. Indeed, over 24% of youths exposed to a traumatic event develop depression, and they are three times more likely to develop a depressive disorder compared to youth without trauma exposure^12,19^. Trauma exposure also increases the specific risk of co-occurring depression and anxiety^14^, a point of particular relevance for transdiagnostic treatments.

Trauma exposure may have important impacts on the efficacy of interventions. Among adolescents with trauma exposure, cognitive behavioral therapy (CBT) failed to separate from control in analyses of three landmark depression trials: the FIRST CBT investigation^20^, the TADS RCT^21^, and the TORDIA study of treatment-resistant depression^22^. Further, within TADS, both CBT and CBT + fluoxetine had diminished effects for trauma-exposed youths, compared to youths without a history of trauma^23^. Surprisingly, childhood trauma exposure remains an unexplored moderator in the large clinical trial literature on therapy for youth anxiety disorders^24^.

Exposure to trauma also has yet to be probed as a moderator in the pediatric transdiagnostic psychotherapy literature. There is some evidence to suggest that transdiagnostic therapy may be effective in treating youth PTSD^25^, but this does not address the question of whether youths with trauma exposure, more broadly, have poorer treatment outcomes than youths who do not have such histories. Further, there has yet to be an investigation that examines the interaction of trauma exposure and depression comorbidity on transdiagnostic treatment outcomes in youths, despite the strong association between trauma exposure, risk for developing depressive disorder alone, and specific risk for developing comorbid anxiety and depression. Depression and trauma exposure are likely tightly interwoven in transdiagnostic treatment samples and may have interactive effects on treatment outcomes. To explore this hypothesis, the current study utilized data from the Brief Behavioral Therapy (BBT) clinical trial, the largest study of transdiagnostic psychotherapy for youth^9^. We explored the main and interactive effects of trauma exposure and depression on the primary registered outcomes of the BBT RCT (clinical response, global functioning, anxiety symptoms, depression symptoms) and on treatment implementation (session attendance, therapy process measures). The BBT trial was implemented in a pediatric primary care setting and drew a diverse pool of community youths, who we expected would have significant exposure to trauma that has been previously unexamined.

## Methods

### Participants

The BBT RCT^9^ enrolled a total of 185 participants, recruited through pediatric primary care clinics primarily by provider referral. Youth were eligible for participation if they met full or probable criteria for an anxiety disorder (i.e., separation anxiety disorder, social anxiety disorder, or generalized anxiety disorder) and/or depression (i.e., major depressive disorder, dysthymia, or minor depression), lived with a consenting legal guardian for at least 6 months, and spoke English. Exclusion criteria included active treatment for anxiety or depression, current suicidality with intent or plan, intellectual disability, psychosis, substance dependence, current child abuse, serious physical illness, and having a diagnosis of PTSD or bipolar disorder. The trial had good retention of participants from baseline, through post-treatment, to follow-up^10^.

In the original sample, only 10 youths met the criteria for depression without a co-occurring anxiety disorder; this cell size was deemed insufficient for inclusion in our planned moderation analyses. Thus, the current archival analyses included a total of 175 youth, after omitting these 10 participants. Demographic data (i.e., age, sex, self-reported race/ethnicity, family income, parent education level, family structure) was collected at baseline via parent report. Participant ages ranged from 8.0 to 16.9 years (*M* = 11.1, SD = 2.5), 57.7% were female, and 21% were Hispanic/Latino. The median family monthly income was $4450; the majority (70.3%) of youth lived with both biological parents, and 63.4% of parents had at least a college degree. The sample was primarily anxious, with 114 youths having one or more anxiety disorders (65%) and 61 youths (34.9%) having both anxiety and depression.

### Procedure

The original trial was approved by the San Diego State University Human Research Protection Program, Kaiser Permanente Southern California Institutional Review Board, and the University of Pittsburgh Institutional Review Board. Participants were recruited from 9 pediatric clinics in San Diego, California and Pittsburgh, Pennsylvania from 2010 to 2014. After being referred by their primary care providers, participants completed a brief telephone screener and were invited to the baseline assessment, which was held in person at the participant’s primary care office. Youth and their parents provided written informed consent and assent at baseline before initiation of study procedures. Participants were evaluated at baseline using the Schedule for Affective Disorders and Schizophrenia for School-Age Children-Present and Lifetime Version (K-SADS-PL)^26^ semi-structured interviews, which were administered by Masters-level independent evaluators (IE) to ascertain clinical diagnoses. Participants were considered clinically depressed if they met the criteria for a depression diagnosis (using DSM-IV criteria) and/or if they obtained a score of 40 or higher on the Children’s Depression Rating Scale-Revised (CDRS-R)^27^. Those who met inclusion criteria for anxiety and/or depression at baseline were then randomly assigned to BBT or ARC, using Begg and Iglewicz’s^28^ modification of Efron’s biased coin toss, which balanced on participant sex, ethnicity, and presence of clinically significant depression. Youth and their caregivers in both conditions completed assessments at baseline, 16 weeks after baseline (i.e., BBT post-treatment), and 32 weeks after baseline. The Week 16 and Week 32 follow-up assessments were administered over the phone by IEs who were masked to participant conditions.

### Interventions

Brief Behavioral Therapy (BBT) is a manualized transdiagnostic psychotherapy that contains elements of established evidence-based treatments for anxiety and depression. BBT was designed to address two core behavioral deficits shared by anxiety and depression by (a) reducing youths’ avoidance of threats and (b) increasing youths’ approach behaviors toward rewarding life experiences. These mechanisms map onto transdiagnostic Research Domain Criteria^29^ (RDoC Negative Valence Systems, Potential and Sustained Threat; RDoC Positive Valence Systems, Reward Learning) and are targeted through the technique of graded engagement, a combination of exposure (anxiety) and behavioral activation (depression) techniques. Compared to CBT monotherapies and other transdiagnostic interventions, BBT is unique in that it does not include a module dedicated to cognitive restructuring. The BBT program consists of 8–12 sessions; in the original trial, youth received an average of 11.2 sessions of BBT. Each session took place in person at a primary care facility, lasted about 45 min, and was administered by a Masters-level therapist. BBT was administered with high adherence, with an average of 96% of the manual content being delivered, based on session recordings assessed to ensure fidelity^9^.

Assisted Referral to Care (ARC) was based on evidence-based methods shown to decrease no-show rates and increase access to outpatient care through referrals^30^. In an initial phone call following the baseline assessment and randomization, Masters-level study staff provided feedback to the consenting caregiver on the youth’s symptoms and basic psychoeducation regarding the importance of addressing symptoms. Referrals were provided to clinicians and community mental health facilities specializing in youth mental health care based on match to family location and insurance status. Caregivers then received a phone call from the study staff every 2 weeks, with a median of four calls administered, to promote follow-through with referral to treatment and problem-solve barriers to accessing care. A total of 82% of ARC families obtained access to treatment, with a mean of 6.5 outpatient sessions attended^9^.

### Trauma exposure

Trauma exposure was collected at baseline from youths and parents as part of the administration of the K-SADS PTSD module^26^. Youth and their parents were asked to respond to a yes/no checklist that detailed a variety of traumatic events (i.e., car accident, other accident which caused injury, fire, natural disaster, a victim of/witness to violent crime, receiving traumatic news, witness to domestic violence, and physical or sexual abuse). If endorsed, subsequent items on the K-SADS PTSD module probing diagnostic criteria for PTSD were administered. Youths who met diagnostic criteria for a current PTSD diagnosis were excluded from the trial and referred for alternative services; youth not meeting diagnostic criteria for PTSD were retained. For moderation analyses, trauma exposure was coded as absent or present (i.e., yes to one or more types of exposure(s) on the PTSD events checklist, per youth or parent report). This method of assessing trauma exposure parallels the methods used in the TORDIA trial^22^ and was similar to the procedures used in the TADS RCT^21^.

### Primary clinical outcomes

Clinical interviews were conducted with youths and parents at baseline, Week 16, and Week 32. Measures were rated by IEs masked to treatment assignment, with good inter-rater reliability across measures (ICC = 0.70–0.95). The Clinical Global Impressions severity scale (CGI-S)^31^ indexed overall clinical severity at baseline; scores range from 1 to 7, with a score of 7 indicating severe impairment, and both CGI-S and Improvement (CGI-I)^31^ were rated at Weeks 16 and 32. A clinically significant response to treatment was defined as a score of ≤2 on the CGI-I. The Children’s Global Assessment Scale (CGAS)^32^ was completed by the IE and rated functioning across school, home, and amongst peers. Scores range from 0 to 100, with scores below 60 being indicative of impaired functioning. The Pediatric Anxiety Rating Scale (PARS)^33^ is a 50-item checklist with seven severity items for anxiety severity in the past week; the clinician combines youth and parent ratings with their clinical judgment based on each interview. The PARS has been shown to have high interrater reliability, adequate internal consistency, and sensitivity to treatment effects. The CDRS-R is a 17-item semi-structured clinician-rated assessment of depression symptoms, with scores of 40 or higher being indicative of clinically elevated depression. The CDRS-R has been shown to have good interrater-reliability and internal consistency, as well as being sensitive to treatment effects^27^.

### Treatment Implementation

In BBT, therapists rated youth engagement, youth homework completion, therapist adherence to the treatment model, and therapeutic alliance at the end of every session on single-item questions ranging from 0 (low) to 4 (high). In both BBT and ARC, the use of non-study outpatient services was assessed by parent- and youth-report on the Child and Adolescent Services Assessment (CASA)^34^.

### Data analytic plan

All statistical analyses utilized SPSS version 28.0. All tests of significance were two-tailed, and alpha was set at *p* < 0.05 for all tests. Adjustments for multiple comparisons were not made due to the hypothesis-generating goal of these analyses. Differences in baseline characteristics across patterns of comorbid depression and trauma exposure were examined using ANOVA for continuous dependent variables and logistic regression, with follow-up chi-square tests, for categorical variables. Statistically significant differences in baseline characteristics across patterns of comorbid depression and trauma exposure were used as covariates in analyses examining moderation.

Analyses were then run to examine the relationship between comorbid clinical depression (presence of depression diagnosis and/or CDRS-R ≥ 40 at baseline) and trauma exposure and their effect on treatment outcomes at specific follow-up time points. Due to non-linear change over time, regression analyses were conducted at Week 16 (post-treatment) and Week 32 (follow-up) assessments separately. To test this, a treatment group (BBT or ARC) by depression status (not depressed vs. depressed) by trauma exposure (no exposure vs. exposed to at least one type traumatic event at baseline) three-way interaction term (i.e., treatment × depression × trauma exposure) was included in logistic regression models for the CGI-I at Week 16 and at Week 32, which also included all lower order interactions and main effects and covariates discussed previously. Since one of the subgroups in the ARC arm had a zero-response cell at Week 16, we added a dummy value to the zero cells in order to be able to run the regression model^35^. The omnibus logistic regression tests were followed by chi-square tests of simple effects. The three-way interaction term was also included in linear regression analyses for the three continuous outcome variables (CGAS, PARS, CDRS-R) at Week 16 and at Week 32. All lower-order interactions and main effects, as well as the baseline measure of the outcome variable as a covariate, were included in the models. Simple slope analyses were run to better understand higher-order effects, with the baseline measure of the outcome variable as a covariate.

Exploratory analyses examining differences in treatment implementation and therapy process by comorbid depression status, trauma exposure, and their interaction, were tested with ANOVA models. Number of sessions attended between baseline and Week 16, Week 16 and Week 32, and across the trial were examined in both ARC and BBT. Therapist adherence, youth engagement, homework completion, and therapeutic alliance were tested among youth in the BBT arm.

## Results

### Baseline characteristics

Nearly half (46.3%; *n* = 81) of youths reported exposure to one or more type(s) of traumatic event(s) at baseline. Among youths with trauma exposure, 48.1% had exposure to more than one type of traumatic event (*M* = 1.89, SD = 1.10). The most common single type of traumatic event was witnessing domestic violence (25.9%), although a broad range of direct exposures were endorsed (e.g., 2.5% sexual abuse; 6.2% victim of/witness to violent crime; 8.6% physical abuse; 14.8% fire; 18.5% natural disaster; 32.1% car/other accident). Rates of trauma exposure and experience of multiple types of traumas did not significantly differ between BBT and ARC (*p* = 0.203; *p* = 0.100).

As expected, there was a substantial overlap between trauma exposure and the presence of comorbid clinically significant depression at baseline. Among youths with trauma exposure, 49.3% had clinically significant depression; in contrast, only 22.3% of youths without reported trauma exposure met the criteria for clinically significant depression at baseline (*p* < 0.001). Note that within the trauma-exposed youth, the presence of comorbid depression was *not* significantly associated with the number of different types of trauma endorsed or with exposure to interpersonal trauma (i.e., domestic violence, physical/sexual abuse, victim/witness to violent crime; all *p* > 0.157). However, among trauma-exposed youth, those with comorbid depression (versus those without) were significantly more likely to be experiencing at least one, current criterion symptom of PTSD at baseline (32.4% vs. 8.1%; *p* = 0.009).

Table 1 displays differences in demographic and clinical characteristics at baseline by presence of trauma exposure and by comorbid depression. As noted, youths with trauma exposure had significantly higher depression severity scores than youths without trauma exposure (*p* < 0.001); in addition, their families reported significantly lower monthly income (*p* = 0.010), and parents were less likely to have a college degree (*p* = 0.010). Youth with comorbid depression evidenced higher severity across all baseline values of clinical outcomes (all *p’s* < 0.001). In addition, they were more likely to be older (*p* < 0.001) and female (*p* = 0.009); their parents also were less likely to have a college degree (*p* = 0.007). A significant depression × trauma exposure interaction was only observed for parental education, driven by the anxious-only without trauma cell, in which parents were significantly *more* likely to have a college degree.

**Table 1:** Baseline characteristics by presence of comorbid depression and trauma exposure.

	Anxious-only without trauma (*n* = 73)	Anxious-only with trauma (*n* = 41)	Anxious-depressed without trauma (*n* = 21)	Anxious- depressed with trauma (*n* = 40)	Depression effect *p-*value	Trauma effect *p*-value	Depression × Trauma *p*-value
*Demographic*							
Age (*M*, SD)	10.15^a^ (1.8)	10.61^a^ (2.7)	12.05^b^ (2.5)	13.00^b^ (2.3)	<0.001	0.061	0.510
Female (%)	42.46%^a^	53.65%^ab^	76.19%^bc^	80.00%^c^	0.009	0.251	0.764
Caucasian (%)	69.86%	65.85%	71.42%	67.50%	0.890	0.659	0.998
Living with both biological parents (%)	82.19%	70.73%	76.19%	45.00%	0.539	0.159	0.344
Parent college graduate (%)	78.87%^a^	55.00%^b^	47.61%^b^	57.50%^b^	0.007	0.010	0.029
Monthly income, median*	5550	3800	6000	3750	0.517	0.010	0.267
*Clinical*							
CGI-S (*M*, SD)	3.85^a^ (0.8)	3.93^a^ (0.6)	4.76^b^ (0.4)	4.60^b^ (0.7)	<0.001	0.710	0.293
CGAS (*M*, SD)	58.73^a^ (6.6)	57.59^a^ (5.9)	51.76^b^ (3.8)	52.10^b^ (6.7)	<0.001	0.698	0.475
PARS 6-item (*M*, SD)	14.18^a^ (4.4)	14.46^a^ (5.1)	17.14^b^ (4.5)	17.20^b^ (4.9)	<0.001	0.827	0.884
CDRS-R (*M*, SD)	23.28^a^ (4.5)	27.88^b^ (6.1)	42.81^c^ (8.2)	46.55^c^ (10.9)	<0.001	<0.001	0.722

ANOVAs were conducted for continuous variables and logistic regression for categorical variables. Superscripts indicate groups that were significantly different from each other on post-hoc tests. Post-hoc tests were only conducted in the presence of significant main and/or interaction effects.

*CGI-S* Clinical Global Impressions-Severity, *CGAS* Children’s Global Adjustment Scale, *PARS* Pediatric Anxiety Rating Scale, *CDRS-R* Children’s Depression Rating Scale-Revised.

*Analyses for income were conducted using a square root transformation in order to run an ANOVA; raw median monthly income in US dollars for each subgroup are provided.

### Tests of moderation by outcome domain

Supplementary Table 1 includes all main effects, lower-order, and higher-order interactions across all four outcome measures. Table 2 provides a summary of these detailed data to give an overview of patterns of results. In the text, we describe the main effects of treatment and the three-way interactions of interest on our four outcome measures. Note that follow-up analyses of significant two-way interactions (i.e., treatment × depression status; treatment × trauma exposure) did not change the interpretation of the higher-order moderation effects. Age, gender, parent education, and family income were included as covariates in analyses testing the sensitivity of moderation effects. Inclusion of covariates did not change the statistical significance of the interactions for any outcome; thus, for simplicity, unadjusted effects are presented in the text and in Supplementary Table 1.

**Table 2:** Summary of significant BBT treatment and moderator effects.

Clinical outcome (time in weeks)	Significant model effect		Significant effects for BBT compared to ARC within subgroup			
	Main effect of tx	Tx × Dep × Trauma	Anx No trauma	Anx + Trauma	Anx/Dep No Trauma	Anx/Dep + Trauma
CGI-I (16)	**✓**	**✓**	**✓**	**✓**	**✓**	ns
CGI-I (32)	**✓**	ns	**✓**	**✓**	ns	ns
CGAS (16)	ns	**✓**	**✓**	**✓**	**✓**	ns
CGAS (32)	ns	**✓**	ns	**✓**	**✓**	ns
PARS (16)	**✓**	ns	**✓**	**✓**	ns	ns
PARS (32)	**✓**	**✓**	**✓**	**✓**	**✓**	ns
CDRS (16)	ns	**✓**	ns	**✓**	**✓**	ns
CDRS (32)	ns	**✓**	ns	ns	**✓**	ns
Total number of significant effects	4	6	5	7	6	0

*Tx* treatment, *Dep* comorbid clinically significant depression at baseline, *Anx* clinically significant anxiety at baseline, *Trauma* endorsed on KSADS, *No Trauma* no trauma endorsed on KSADS, *CGI-I* Clinical Global Impressions-Improvement, *CGAS* Children’s Global Adjustment Scale, *PARS* Pediatric Anxiety Rating Scale, *CDRS-R* Children’s Depression Rating Scale-Revised, **✓** statistically significant, *ns* non-significant.

### Treatment response (CGI-I)

**Fig. 1:**
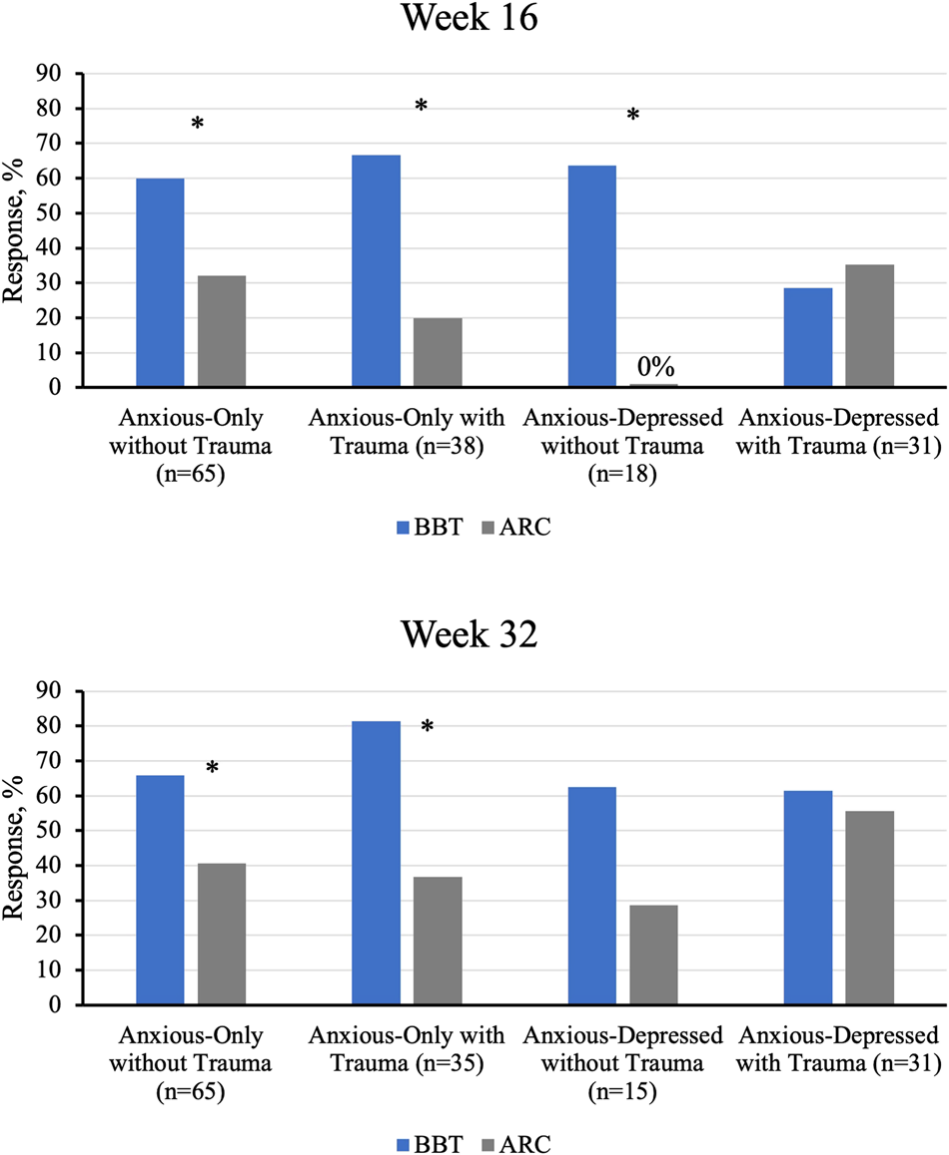
Response rates at Week 16 and Week 32. Response was defined as a Clinical Global Impressions-Improvement (CGI-I) score ≤ 2. *p < 0.05 for comparison of BBT and ARC.

The overall model fit for the logistic regressions predicting Week 16 and Week 32 CGI-I were both good (all Hosmer–Lemeshow *p’*s > 0.05). For Week 16 CGI-I, analyses found a significant main effect of the treatment group favoring BBT (*p* = 0.031) as well as a significant treatment × depression × trauma interaction (*p* = 0.031). Chi-square tests were used to probe the three-way interaction. As seen in Fig. 1, within the group of anx/dep youth *with* trauma exposure, the previously significant effect of BBT on the CGI-I at post-treatment was no longer present (28.6% in BBT vs. 35.3% in ARC; *p* = 0.690). However, significant treatment effects were found favoring BBT for the other three clinical subgroups: anxious-only youth without trauma exposure (60.0% in BBT vs. 32.0% in ARC; *p* = 0.028), anxious-only youth with trauma exposure (66.7% in BBT vs. 20.0% in ARC; *p* = 0.004), and anx/dep youths without trauma exposure (63.6% BBT youths and 0% of ARC; *p* = 0.007).

At Week 32, the logistic regression model predicting treatment response found a significant main effect of treatment (*p* = 0.048), favoring BBT. The three-way interaction at Week 32, however, was not statistically significant (*p* = 0.188). Exploratory follow-up chi-square tests were conducted to examine treatment effects within each sample subgroup. Response rates were significantly higher in BBT for two of the four clinical subgroups: anxious-only youth without trauma exposure (65.8% in BBT vs. 40.7% in ARC; *p* = 0.045) and anxious-only youth with trauma exposure (81.3% in BBT vs. 36.8% in ARC; *p* = 0.008). Response rates did not significantly differ between treatment groups among anx/dep youth without trauma exposure (*p* = 0.189) or anx/dep youth with trauma exposure (*p* = 0.739; see Fig. 1).

### Global functioning (CGAS)

**Fig. 2:**
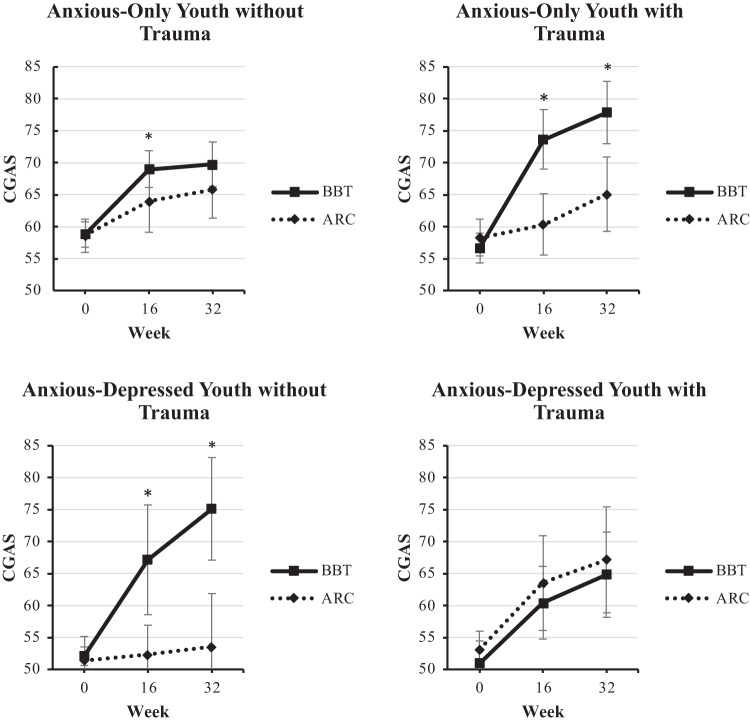
Change in global functioning over time. Week 16 and 32 outcomes are modeled in separate regression analyses, controlling for baseline. Unadjusted raw means are presented. *p < 0.05 for comparison of BBT and ARC. Error bars indicate 95% confidence intervals.

At Week 16, the linear regression model predicting functioning did not show a main effect for BBT treatment (*p* = 0.055); however, there was a significant three-way interaction (*p* < 0.001). Simple slope analyses were conducted to probe the three-way interaction, including baseline functioning as a covariate for each analysis. As seen in Fig. 2, the analyses found a significant treatment effect in three of the four clinical subgroups under investigation. BBT was significantly superior to ARC amongst anxious-only youth with trauma exposure (*p* < 0.001) and without trauma exposure (*p* = 0.048) and among anx/dep youth without trauma exposure (*p* = 0.013). In contrast, no significant treatment effect for BBT was found for the subgroup of anx/dep youth *with* trauma exposure *(p* = 0.727).

At Week 32, the linear regression model again resulted in a significant three-way interaction (*p* < 0.001), with no main effect for treatment in this expanded model (*p* = 0.185). As seen in Fig. 2, BBT continued to be significantly superior to ARC in two of the four subgroups, anxious-only youth with trauma (*p* < 0.001) and anx/dep youth without trauma (*p* = 0.001). The previously significant effect of BBT at Week 16 for anxious-only youth without trauma was no longer significant at Week 32 (*p* = 0.173). Additionally, as was seen at Week 16, BBT did not statistically separate from ARC in anx/dep youth *with* trauma (*p* = 0.670).

### Anxiety (PARS)

**Fig. 3:**
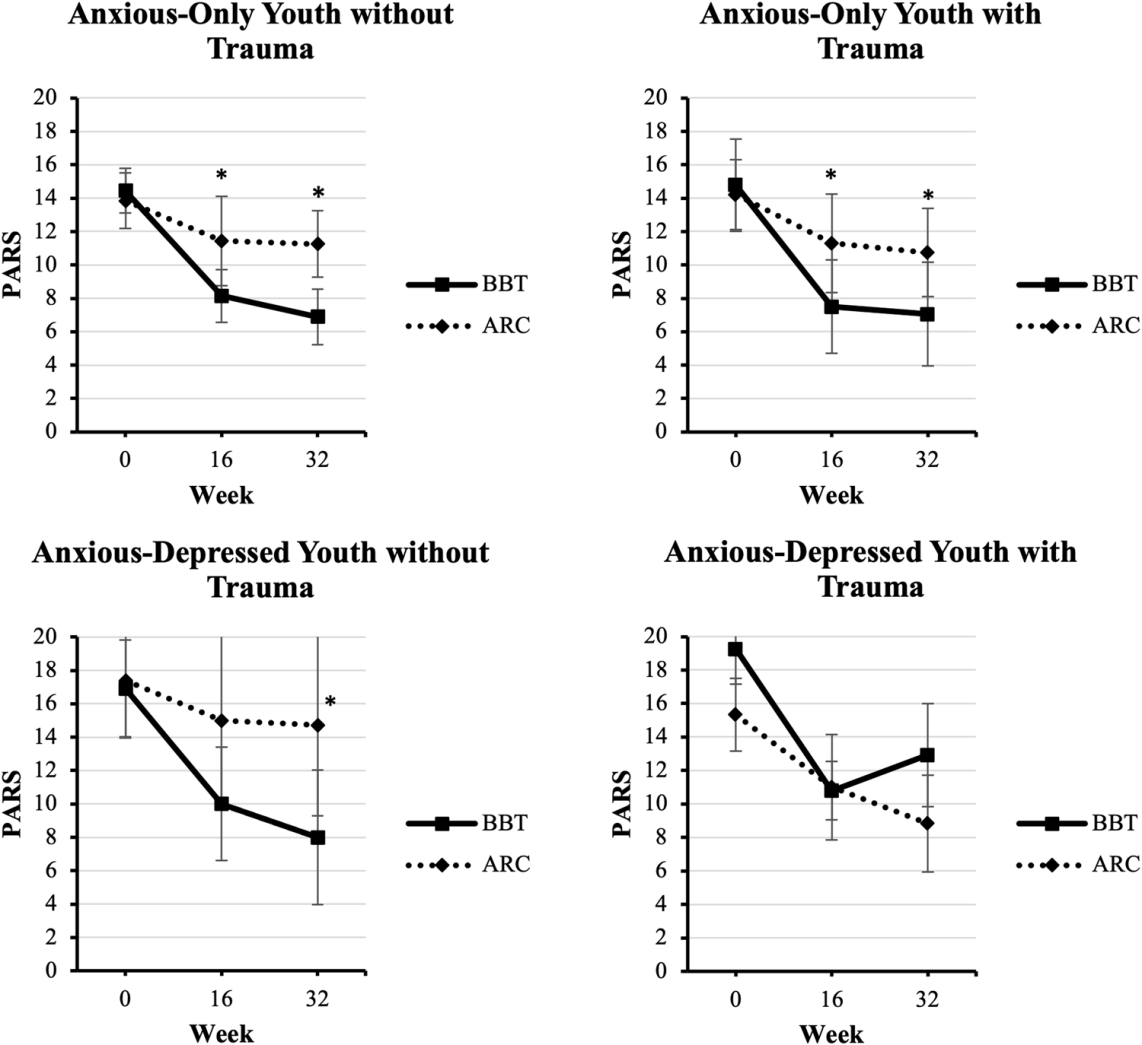
Change in anxiety over time. Week 16 and 32 outcomes are modeled in separate regression analyses, controlling for baseline. Unadjusted raw means are presented. *p < 0.05 for comparison of BBT and ARC. Error bars indicate 95% confidence intervals.

The regression model predicting anxiety resulted in a significant main effect of treatment (*p* = 0.013), with youth in BBT having significantly lower anxiety scores than ARC youth at post-treatment (Week 16). No significant three-way interaction was found at Week 16 (*p* = 0.251). Exploratory follow-up tests were conducted to examine treatment effects within each of the sample subgroups, with significant effects in two of the four groups. BBT was superior to ARC among anxious-only youth with (*p* = 0.037) or without trauma exposure (*p* = 0.018) at Week 16 (see Fig. 3). In contrast, there were no significant differences between treatment groups among anx/dep youth without trauma exposure (*p* = 0.068) or with trauma exposure (*p* = 0.843).

At Week 32, the regression model predicting anxiety scores resulted in a significant main effect of treatment (*p* < 0.001), favoring BBT over ARC, as well as a significant three-way interaction (*p* = 0.028). Simple slope analyses were then conducted, including baseline anxiety as a covariate for each analysis. As shown in Fig. 3, there was again a significant effect of the treatment group in three of the four subgroups. BBT youth had significantly lower anxiety scores at Week 32 compared to youth in ARC for anxious-only youth with no history of trauma exposure (*p* < 0.001), anxious-only youth with trauma exposure (*p* = 0.007), anx/dep youth with no reported exposure to trauma (*p* = 0.043). BBT and ARC did not significantly separate in the group of anx/dep youth *with* trauma (*p* = 0.156) (see Fig. 3).

### Depression (CDRS-R)

**Fig. 4:**
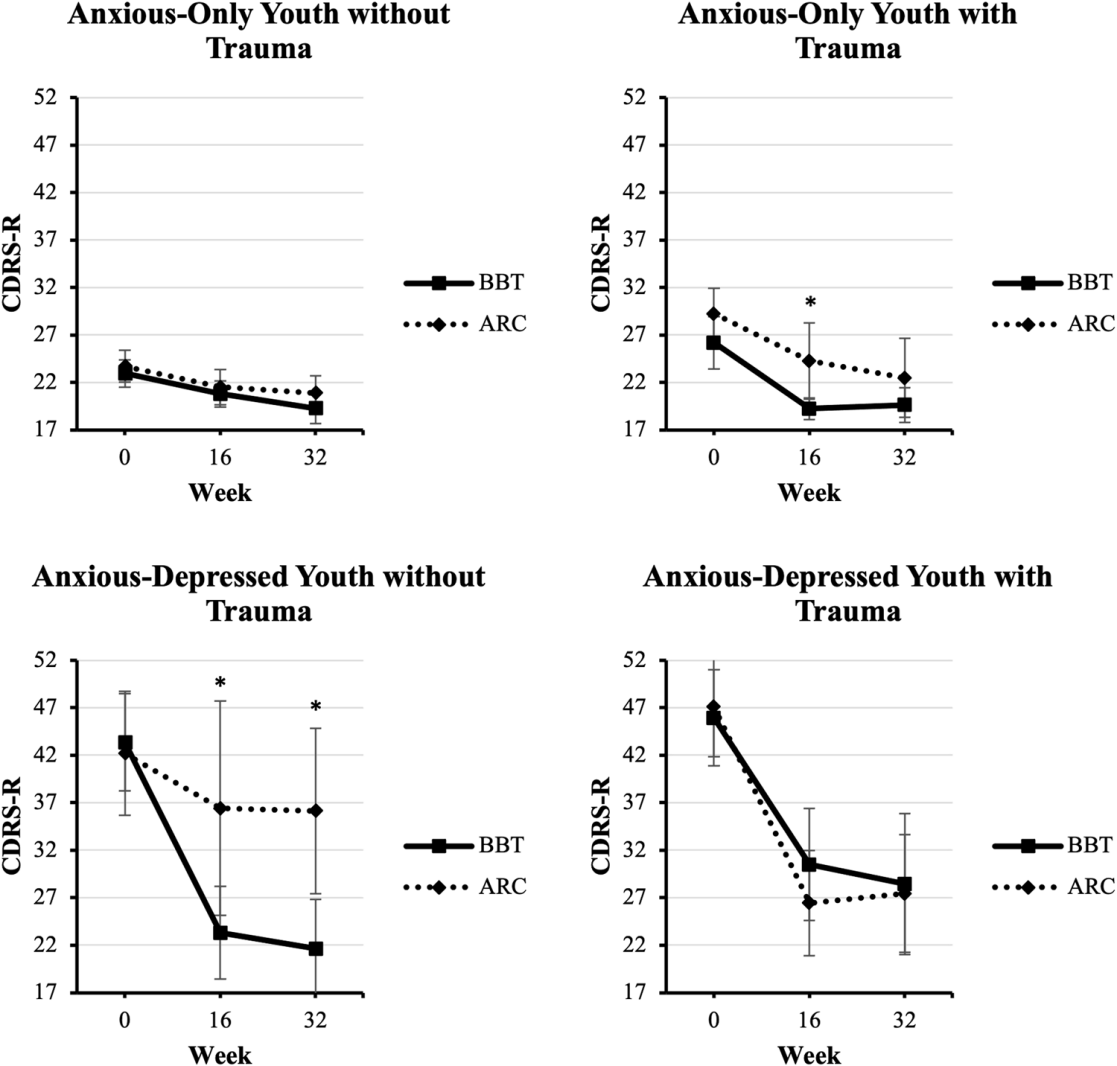
Change in depression over time. Week 16 and 32 outcomes are modeled in separate regression analyses, controlling for baseline. Unadjusted raw means are presented. *p < 0.05 for comparison of BBT and ARC. Error bars indicate 95% confidence intervals.

As can be seen in Fig. 4, CDRS-R scores were low across time for youths who did not meet the criteria for comorbid clinically significant depression at baseline. Nevertheless, at Week 16, the regression model predicting depression scores resulted in a significant three-way interaction (*p* < 0.001), with no main effect for treatment (*p* = 0.782). In simple slopes analyses, BBT was found to be significantly superior to ARC for two of the four clinical subgroups of youth, anxious-only youth with trauma exposure (*p* = 0.049) and anx/dep youth without trauma exposure (*p* = 0.015). No significant difference between treatment groups was found amongst anxious-only youth without trauma exposure (*p* = 0.712) nor amongst anx/dep youth *with* trauma exposure (*p* = 0.290).

At Week 32, the regression model predicting depression resulted in a significant three-way interaction (*p* = 0.001); no significant main effect of treatment was found (*p* = 0.489). Follow-up analyses found a significant treatment effect favoring BBT on depression symptoms amongst anx/dep youth without trauma exposure (*p* = 0.005). BBT did not significantly separate from ARC at Week 32 in anxious-only youth without trauma exposure (*p* = 0.243), anxious-only youth with trauma exposure (*p* = 0.353), or anx/dep youth *with* trauma exposure (*p* = 0.462).

### Treatment implementation

In a final set of exploratory analyses, we examined whether differences in the number of sessions attended or treatment process factors explained the observed moderation findings. Note that in our previous work, the number of sessions attended did not predict outcome within ARC^10^, and BBT was superior to ARC in cost-effectiveness when adjusting for the higher cost/dose of BBT relative to clinical benefit^36^. Analyses in this section were designed to be hypothesis-generating and provide guidance on future treatment development to enhance the outcomes of our most vulnerable group of youths (i.e., those with anxiety, depression, and trauma exposure).

Supplementary Table 2 provides descriptive statistics and inferential tests of the depression × trauma exposure interaction within each treatment group on relevant variables. Within ARC, there were no significant main or interactive effects of depression status or trauma exposure on the number of sessions attended from baseline to Week 16, Week 16 to Week 32, or over the course of the trial (baseline-Week 32; all *p* > 0.129). Exploratory pairwise comparisons within ARC indicated that anx/dep youth with trauma exposure had significantly more outpatient treatment sessions than anxious-only youths without trauma exposure (baseline to Week 16, *p* = 0.019; baseline to Week 32, *p* = 0.036); no other pairwise comparison yielded significant results. Within BBT, there was an effect of depression status on the number of BBT sessions; youths with comorbid depression attended fewer sessions on average than youths without (10.59 vs. 11.68 sessions; *p* = 0.019). There were also significant differences in the use of outpatient services after the conclusion of BBT (Week 16–Week 32), driven by a significant depression × trauma exposure interaction (*p* = 0.028). Pairwise comparisons indicated that anx/dep youth *with* trauma exposure obtained significantly more (*M* = 3.80, SD = 4.61) sessions of outpatient care after the conclusion of BBT than youths in all other subgroups enrolled in BBT (all *p* ≤ 0.007). As seen in Supplementary Table 2, there were no significant effects of depression, trauma exposure, or their interaction on BBT therapy process variables including therapist adherence to the BBT treatment model, child engagement, homework completion, or therapeutic alliance (all *p* > 0.324).

## Discussion

The public health rationale for the development of transdiagnostic treatments is based, in large part, on their purported efficiency. Compared to disorder-specific monotherapies, transdiagnostic interventions bring a unified and coherent approach to youths struggling with an interconnected web of internalizing symptoms and provide a single, comprehensive treatment model for providers and payors seeking to simplify the task of implementing evidence-based practices across disorders. However, little is known about whether the positive effects of transdiagnostic interventions are shared equally across different patterns of depression comorbidity and for those with a history of trauma exposure.

Results of the current investigation provide a great deal of good news. Overall, the transdiagnostic BBT treatment was effective for three of the four clinical subgroups probed in this study. Indeed, the current moderator analyses clarified and improved upon findings from the original BBT trial. In the original paper, BBT did not evidence a main effect on depression symptoms^9^, and, in analyses of follow-up data, the superior effect of BBT on anxiety outcomes was not seen for youths with clinically significant depression at baseline^10^. The current analyses untangled depression and trauma exposure and led to more positive findings for more clinical subgroups of youths. BBT treatment effects on depression symptoms were found for two of the four subgroups of youths, and comorbid depression with anxiety (but without trauma) did not significantly moderate anxiety outcomes. Further, we uncovered BBT treatment effects on anxiety, depression, functioning, and response to treatment for youths with trauma exposure—adding to the very sparse literature examining this understudied, but widely prevalent, potential moderator. In general, the BBT model appeared to be robust for anxious youth with clinically significant depression *or* with childhood trauma exposure.

Despite this good news, troubling results were found for anxious youths with *both* clinically significant depression at baseline and childhood trauma exposure. Moderator analyses identified this subgroup as a clinically “vulnerable cell” that encompassed nearly a quarter of the enrolled sample (22.8%, *n* = 40). As seen in Table 2, in the other clinical subgroups of youths, BBT was superior to ARC on most outcomes at most time points. In contrast, BBT *never* significantly separated from ARC in the subgroup of anxious youths with both comorbid depression and trauma exposure. Failure of BBT to separate from ARC could logically result from (a) dampened effects of BBT in this subgroup, (b) enhanced effects in ARC, (c) “equivalent” positive treatment effects in both BBT and ARC that could not be statistically differentiated, or (d) “equivalent” failure to show treatment effects in both BBT and ARC. As can be seen in the figures, improvement in symptoms and functioning did occur for anxious youths with depression and trauma exposure, but the magnitude of this change was similar in both BBT and ARC. For anxiety and depression outcomes, this pattern may suggest that youths in both groups experienced positive treatment effects, as mean scores for follow-up assessments in both BBT and ARC approached or passed into the normal range^33,37^. However, examining clinical response on the CGI-I tells a different tale. For anxious youths with depression and trauma exposure, BBT and ARC did not significantly differ at follow-up assessments, and the overall response rates were quite low. Indeed, at Week 16, only 28.6% of youths in BBT and 35.3% of youths in ARC were rated as clinically improved, rates that are comparable to the pill placebo control conditions in benchmark efficacy trials for youth anxiety (23.7%)^38^ and adolescent depression (34.8%)^39^. Clinical response rates for youths with depression and trauma exposure improved in both BBT and ARC at long-term follow-up. However, youths with depression and trauma in BBT were significantly more likely to receive additional care during this follow-up interval than youths in other subgroups. Similarly, youths with depression and trauma in ARC had significantly more sessions through 32 weeks, supporting the interpretation that this subgroup of youths was not clinically recovered at acute follow-up. Taken together, our data suggest that youths with anxiety in combination with depression and trauma exposure may be a vulnerable group at risk for poorer outcomes.

The mechanism underlying poor effects for anxious youths with depression and trauma exposure is unfortunately not clear. Youths with comorbid depression and anxiety were considerably different from anxious-only youth at baseline (i.e., more likely to be older, female, had higher scores on dimensional measures of both anxiety and depression, poorer functioning, greater overall clinical severity, and were less likely to have parents with at least a college degree). Youth with a history of trauma were also considerably different than youth without trauma exposure at baseline; they had greater depression symptoms, lower monthly household income, and their parents were less likely to have at least a college degree. However, despite these significant differences, there were no changes in the pattern of results across clinical outcomes when baseline characteristics were included as covariates in analytic models. These differences may be markers for other processes that may provide explanatory power in future research, such as greater length of disorder or chronicity, but these data were unavailable in the current report.

To further probe possible mechanisms of our moderation effect, we examined whether implementation of and engagement in treatment varied by depression status and trauma exposure. As in the Brent et al. ^10^ analyses, youths with depression and anxiety attended fewer sessions of BBT on average than anxious-only youths; however, the depression × trauma exposure interaction was not significant for BBT session attendance. In contrast, a significant depression × trauma exposure interaction was found for the use of outside mental health services for youth in the BBT arm. Compared to all other subgroups, anxious youth with both depression and trauma exposure attended a greater number of outpatient sessions after the conclusion of BBT (between weeks 16 and 32). These data could reflect the poorer outcomes of youths in this cell (i.e., lower acute rates of clinical improvement) and the need for additional sessions to consolidate skills or, alternatively, the desire to shift treatment focus to other clinical issues outside of the transdiagnostic protocol. Within ARC, there also was evidence that anxious youths with depression and trauma exposure received significantly more outpatient sessions when compared to “anxious only” youths (those without depression and without trauma), perhaps indicating a similar need for additional or extended services. Within BBT, there were no main or interactive effects of depression or trauma exposure on any of the process indicators rated by therapists, including therapeutic alliance, child engagement, and homework completion, or therapist adherence to the treatment model. These process factors have been hypothesized by others^23^ to be the source of the negative moderation effects of trauma seen in CBT studies for adolescent depression. We did not find evidence for this in our sample with our process measures.

Looking beyond our data, the neuroimaging, psychobiological, and learning literature may provide hints about how treatment may be impacted by comorbid depression and trauma in our sample of anxious youth. For instance, there is evidence that brain structure and function in areas implicated in learning and memory consolidation may differ between individuals with both depression and a history of trauma compared to those with depression or trauma alone^40,41^. These findings suggest that the combination of depression and trauma exposure could be associated with unique patterns of learning that may call for adapted intervention strategies or for an extended dose of intervention to consolidate gains. Indeed, lengthening treatment for this subgroup of youth or adopting a stepped care model would be in line with the pattern of service use seen in our data: youths with anxiety, depression, and trauma sought out and attended significantly more sessions of non-protocol services after the termination of BBT, and, within ARC, these youths had the highest level of service use across time points. Trauma history and depression may also impact the efficacy of exposure techniques. Theories of behavioral treatment mechanisms for anxiety emphasize that new, non-fearful associations learned in treatment should occur in the context of psychophysiological arousal for new learning to occur (i.e., that the situation is safe and that the individual can tolerate distress associated with symptoms). However, while anxiety has been associated with psychophysiological and biological elevations at rest and reactivity during stress, both trauma exposure and depression have been associated with psychophysiological and biological blunting^42,43^. If trauma exposure and depression each result in blunting, this might theoretically reduce the effectiveness of exposure-based learning on anxiety outcomes for the subgroup of youth with both factors, in an additive or in a multiplicative fashion. In a similar vein, the experience of early adversity in childhood may be associated with changes in sensitivity to reward and punishment, with implications for the effectiveness of behavioral activation techniques. Data are mixed on the relative contribution of early trauma versus neglect and deprivation to disturbances in reward processing^44^. However, there is some evidence that adults with a history of childhood interpersonal trauma report reduced motivation for reward and enhanced sensitivity to punishment, compared to adults without childhood trauma, and that reduced reward motivation is specifically associated with depression symptoms^45^. The central element of BBT—graded engagement—combines both exposure and behavioral activation to reduce avoidance of stimuli associated with negative affect and to increase the approach to reward. Across these studies, a useful hypothesis emerges for future investigation: Do youths with anxiety, depression, and a history of trauma exposure “learn less” from the behavioral lessons of treatment than youths who do not have this cluster of characteristics?

We also see benefits in future work aimed at testing implementation models designed to move transdiagnostic treatments into regular clinical practice. BBT worked well for three of the four clinical subgroups under investigation, and, while BBT did not separate from ARC for anxious youths with depression and trauma exposure, BBT was never significantly inferior to ARC, in any analysis. ARC effects were especially weak for anxiety outcomes, with post-treatment means on the PARS failing to leave the clinical range for all four subgroups of youths. Data were not available on the specific techniques employed in ARC, although community surveys of practitioners suggest that the use of evidence-based therapy for anxious youth, and specifically the use of exposure, is very low^2^. Further, in our sample, anxious-only youth without trauma exposure had the fewest number of outpatient treatment sessions in ARC, receiving almost half of the number of sessions as youths with anxiety, depression, and trauma exposure (4.3 sessions vs. 7.7 at Week 16). These results are in line with national data documenting the undertreatment of pediatric anxiety disorders, with service uptake rates even lower than those for depression^1^, and highlight the need for the dissemination of brief, effective intervention models.

Although the sample size from this transdiagnostic RCT was large relative to other trials, our exploratory analyses of three-way interactions were likely underpowered, and we did not pursue within-treatment analyses, given the small cell sizes. The aims of this study were hypotheses generating, and analyses did not correct for multiple comparisons, with this goal in mind. We were reassured, somewhat, by the consistency of our findings across measures suggesting that youths in the “vulnerable cell” were quite distinct from youths in the other subgroups we examined. Replication of our specific findings seems warranted, especially as the possible mechanisms for the observed effects may have implications for both monotherapies for anxiety and depression as well as for transdiagnostic protocols. Anxiety and depression have very high rates of co-occurrence, and trauma exposure may be an unmeasured and understudied factor influencing treatment success across trials with a variety of foci. Sample size also limited analyses in the current study to a simple trauma exposure marker (yes/no), rather than a more nuanced examination of moderation by trauma type or a more complete assessment of subsyndromal PTSD symptoms. We did not find differences in the type of trauma experienced or in the number of *different* types of trauma experienced by youths with and without comorbid depression in our sample. However, our use of the K-SADS PTSD section as a screening tool limited our ability to assess whether youths experienced multiple traumas within type (e.g., repeated exposure to interpersonal violence) or timing of traumatic events. Larger studies may find value in unpacking trauma in a more fine-grained fashion in the future, including examining the impact of symptoms of traumatic stress rather than simply documenting trauma exposure or PTSD diagnostic status. Encouraging RCTs to report the presence of a trauma history, types of trauma exposure, and symptoms of traumatic stress for youths in their samples would be a good initial step toward developing a larger evidence base.

Overall, the results of the current investigation underscore both the broad effectiveness of the BBT transdiagnostic intervention and the importance of understanding person-level factors related to treatment response. Although there were differences in outcomes across the patterns of comorbid depression and trauma exposure, the significant positive effect of BBT over ARC was generally robust, except for youths who were anxious, depressed, and endorsed a history of trauma exposure. This is a critically important subgroup of youths, given the high prevalence of trauma exposure in the community, the strong overlap between trauma exposure and the presence of depression, and the high comorbidity of anxiety and depression. Future research should work to untangle the mechanisms of this effect to develop more effective intervention models for these high-risk children and adolescents.

### Supplementary information

Supplementary Tables

## Data Availability

Upon request, a minimal dataset will be provided to allow for replication and interpretation of the findings reported in this article.
