# Supplementary material for: Impact of trauma exposure and depression comorbidity on response to transdiagnostic behavioral therapy for pediatric anxiety and depression
Source: Npj Ment Health Res. 2024 Feb 27;3:8. doi: 10.1038/s44184-023-00049-4 (PMC10955846; doi:10.1038/s44184-023-00049-4)
Supplement: Supplementary file 1 — Supplementary Tables [file 44184_2023_49_MOESM1_ESM.pdf]

Supplementary Table 1

Estimates of Logistic Regression models for CGI-I and Linear Regression for CGAS, PARS, and CDRS-R at Week 16 and Week 32

	**Nagelkerke/R^2**	***p***	**b**	***SE***	***p***	**Wald**	**OR**
**CGI-I Week 16**	.178	.003					
Treatment			-1.16	.54	.031	4.67	.31
Depression			.15	.71	.827	.05	1.17
Trauma			.29	.59	.629	.23	1.33
Treatment *x* Depression			-1.35	1.35	.319	.99	.26
Treatment *x* Trauma			-.92	.92	.318	.99	.39
Depression *x* Trauma			-1.76	1.05	.092	2.84	.17
Treatment *x* Depression *x* Trauma			3.74	1.73	.031	4.66	41.93
**CGI-I Week 32**	.121	.054					
Treatment			-1.03	.52	.048	3.91	.36
Depression			-.14	.81	.859	.03	.87
Trauma			.81	.73	.263	1.25	2.25
Treatment *x* Depression			-.39	1.23	.745	.11	.67

Treatment *x* Trauma			-.98	.95	.305	1.05	.38
Depression *x* Trauma			-.85	1.18	.469	.53	.43
Treatment *x* Depression *x* Trauma			2.16	1.64	.188	1.73	8.65
**CGAS Week 16**	.339	< .001					
Treatment			-4.83	2.49	.055		
Depression			3.08	3.41	.368		
Trauma			6.22	2.76	.026		
Baseline CGAS			.73	.13	< .001		
Treatment *x* Depression			-9.47	5.29	.076		
Treatment *x* Trauma			-10.28	4.03	.012		
Depression *x* Trauma			-13.53	4.78	.005		
Treatment *x* Depression *x* Trauma			26.55	7.08	< .001		
**CGAS Week 32**	.279	< .001					
Treatment			-3.64	2.73	.185		
Depression			9.07	4.31	.037		
Trauma			9.43	3.25	.004		

Baseline CGAS			.63	.15	< .001
Treatment *x* Depression			-16.99	6.24	.007
Treatment *x* Trauma			-11.37	4.66	.016
Depression *x* Trauma			-19.43	5.85	.001
Treatment *x* Depression *x* Trauma			34.13	8.30	< .001
**PARS Week 16**	.142	.004			
Treatment			3.51	1.39	.013
Depression			1.27	1.87	.500
Trauma			-.73	1.55	.639
Baseline PARS			.24	.09	.018
Treatment *x* Depression			1.60	2.98	.591
Treatment *x* Trauma			.46	2.25	.838
Depression *x* Trauma			1.24	2.69	.646
Treatment *x* Depression *x* Trauma			-4.61	3.99	.251
**PARS Week 32**	.292	< .001			
Treatment			4.68	1.25	< .001

Depression			.38	1.94	.846
Trauma			-.02	1.48	.992
Baseline PARS			.42	.09	< .001
Treatment *x* Depression			1.36	2.86	.635
Treatment *x* Trauma			-.42	2.09	.842
Depression *x* Trauma			3.95	2.68	.142
Treatment *x* Depression *x* Trauma			-8.44	3.79	.028
**CDRS-R Week 16**	.323	< .001			
Treatment			.49	1.77	.782
Depression			-2.59	2.99	.389
Trauma			-2.37	1.96	.228
Baseline CDRS-R			.25	.09	.005
Treatment *x* Depression			13.04	3.81	< .001
Treatment *x* Trauma			3.96	2.84	.165
Depression *x* Trauma			9.29	3.44	.008
Treatment *x* Depression *x* Trauma			-21.41	5.07	< .001

**CDRS-R Week 32**	.352	< .001			
Treatment			1.27	1.83	.489
Depression			-5.47	3.30	.100
Trauma			-.97	2.18	.656
Baseline CDRS-R			.39	.09	< .001
Treatment *x* Depression			13.28	4.16	.002
Treatment *x* Trauma			.94	3.06	.758
Depression *x* Trauma			7.63	3.91	.053
Treatment *x* Depression *x* Trauma			-17.88	5.50	.001

Note. CGI-I, Clinical Global Impressions-Improvement; CGAS, Children's Global Adjustment Scale; PARS, Pediatric Anxiety Rating Scale; CDRS-R, Children's Depression Rating Scale-Revised.

Supplementary Table 2

Differences in Therapy Process and Implementation by Presence of Comorbid Depression and Trauma Exposure

	**Anxious-Only without Trauma** (n = 73)	**Anxious-Only with Trauma** (n = 41)	**Anxious-Depressed without Trauma** (n = 21)	**Anxious-Depressed with Trauma** (n = 40)	Depression Effect *p*-value	Trauma Effect *p*-value	Depression *x* Trauma *p*-value
**BBT Implementation**							
Mean BBT Sessions	11.82[a]	11.56[a]	11.18[ab]	10.00[b]	.019	.119	.321
Mean Outpatient Sessions Week 0-16	0.45	1.56	0.18	0.94	.460	.120	.772
Mean Outpatient Sessions Week 16-32	0.56[a]	0.61[a]	1.00[a]	3.80[b]	.004	.023	.028

Mean Outpatient Sessions Week 0-32	1.00[a]	2.17[ab]	1.09[a]	4.29[b]	.227	.019	.268
Mean Total Sessions Week 0-16	11.98[ac]	13.11[a]	11.36[ac]	10.32[c]	.045	.958	.197
Mean Total Sessions Week 0-32	12.52	13.72	12.27	13.32	.775	.332	.947
**BBT Therapy Process**							
Mean Therapist's Adherence to Therapy Model	2.32	2.36	2.24	2.20	.324	.987	.748
Mean Child Engagement with Therapeutic Process	2.72	2.69	2.64	2.49	.384	.585	.680
Mean Child Homework Completion	2.31	2.31	2.27	2.19	.674	.845	.858
Mean Therapeutic Alliance	2.34	2.28	2.15	2.19	.337	.925	.745

**ARC Implementation**							
Mean Outpatient Sessions Week 0-16	4.34	6.67	7.22	7.74	.129	.274	.485
Mean Outpatient Sessions Week 16-32	2.61	4.53	6.29	4.95	.185	.851	.291
Mean Outpatient Sessions Week 0-32	6.47	10.76	12.11	11.48	.135	.387	.245

Note. Superscripts indicate groups that were significantly different from each other on post-hoc tests. Post-hoc tests were only conducted in the presence of significant main and/or interaction effects.
